# Short term adherence tool predicts failure on second line protease inhibitor-based antiretroviral therapy: an observational cohort study

**DOI:** 10.1186/s12879-014-0664-3

**Published:** 2014-12-04

**Authors:** Richard Court, Rory Leisegang, Annemie Stewart, Henry Sunpath, Richard Murphy, Philip Winternheimer, Mashuda Ally, Gary Maartens

**Affiliations:** Department of Medicine, University of Cape Town, Cape Town, South Africa; Division of Clinical Pharmacology, Department of Medicine, University of Cape Town, Cape Town, South Africa; Infectious diseases unit, Nelson Mandela School of Medicine, University of Kwazulu-Natal, Durban, South Africa; Department of Medicine, Albert Einstein College of Medicine, Bronx, New York USA; St. Vincent Hospital, University of Evansville, Indianapolis, Indiana USA; Nelson Mandela School of Medicine, University of Kwazulu-Natal, Durban, South Africa; Doctors Without Borders, New York, USA

**Keywords:** HIV, Second line antiretroviral therapy, Medication adherence, Virologic failure, Pharmacy refill

## Abstract

**Background:**

Most patients who experience virologic failure (VF) on second line antiretroviral therapy (ART) in low-middle income countries fail due to poor adherence rather than antiretroviral resistance. A simple adherence tool designed to detect VF would conserve resources by rationally limiting need for viral load (VL) testing and, in those countries with access to third line ART, the need for resistance testing.

**Methods:**

We conducted an observational cohort study of patients who initiated second line ART at a clinic in Kwazulu-Natal, South Africa. Using clinical and pharmacy refill data extracted from the clinic’s electronic database, we determined risk factors for VF. Three different methods of calculating short term pharmacy refill adherence were evaluated and compared with long term adherence since second line initiation. We also explored the ability of differing durations of short term pharmacy refill to predict VF on second line ART.

**Results:**

We included 274 patients with a median follow up of 27 months on second line ART. VF ranged between 3% and 16% within each six month interval after initiating second line ART. 243 patients with at least one VL after 4 months on second line were analysed in the statistical analysis. Pharmacy refill adherence assessed over shorter periods (4 to 6 months) predicted virologic suppression as well as pharmacy refill assessed over longer periods. The risk of VF fell 73% with each 10% increase in adherence measured from pharmacy refills over a 4 month period. Low CD4 count at second line ART initiation was a significant independent risk factor for VF.

**Conclusion:**

Patients identified as poorly adherent by short term pharmacy refill are at risk for VF on second line ART. This pragmatic adherence tool could assist in identifying patients who require adherence interventions, and help rationalize use of VL monitoring and resistance testing among patients on second line ART.

**Electronic supplementary material:**

The online version of this article (doi:10.1186/s12879-014-0664-3) contains supplementary material, which is available to authorized users.

## Background

Approximately 500 000 people in low-middle income countries were estimated to be on second line protease inhibitor (PI)-based ART in 2012, and this number is expected to increase exponentially [1]. A systematic review reported virologic failure (VF) rates on second line ART in resource-limited settings to be as high as 38% after 3 years [2]. Risk factors for VF on second line ART include a lower CD4 count and the presence of WHO clinical stage 4 conditions at second line switch, [3]-[6], a longer duration between confirmed VF on first line and initiating second line, [7] changing one nucleoside/nucleotide reverse transcriptase inhibitor (NRTI) instead of two at second line ART initiation, [8] and poor adherence on second line ART [9]. The majority of patients with VF on second line ART have no major PI mutations, indicating that VF in most cases is not the result of antiretroviral resistance [10]-[12]. Identifying which patients on second line ART are at risk of developing VF will allow earlier implementation of adherence interventions and may rationalize the use of VL monitoring, which is not widely available in many low-income countries. The development of policies for a third line ART regimen is regarded as a priority by the World Health Organization (WHO) [13]. Empirically switching patients failing second line ART to third line ART, which is currently expensive, would be a waste of resources as most patients do not have antiretroviral resistance. Switching to third line ART should ideally be guided by genotype antiretroviral resistance testing (GART), but this is currently expensive and has little availability in resource limited settings. A simple predictor of VF on second line ART could also be useful in selecting patients who may benefit from GART.

Pharmacy refill is an adherence measure, which has been shown to correlate well with survival and virologic suppression [14]-[16]. A recent study from our group (of an earlier cohort from the same clinic from which the cohort used in the current study was obtained) showed that adherence measured by pharmacy refill correlated with virologic suppression on second line ART, but this was based on average adherence since starting second line [9]. A more pragmatic measure would be to assess adherence over the short term, which has been shown to predict VF in a small study in a high income country setting [17].

We investigated the association between short term adherence, assessed by pharmacy refill, and VF in patients on second line ART at a clinic in Kwazulu-Natal, South Africa, in order to develop a clinical tool for use in ART programmes in low-middle income settings.

## Methods

### Study population and setting

The McCord Hospital ART clinic, “Sinikithemba” meaning in Zulu “we give hope”, provided HIV care for patients from Durban and surrounding areas in Kwazulu-Natal, South Africa with financial support from the President’s Emergency Plan for AIDS Relief and the South African Department of Health. Approximately 8200 adults and 1200 children received ART at the clinic until its closure in February 2012. The clinic followed national recommendations, which at the time included VL and CD4 count monitoring six-monthly after ART initiation. During the period of study, the standard South African second line regimen consisted of lopinavir/ritonavir with two NRTIs. As per WHO guidelines, second line initiation occurred either for toxicity/intolerability of first line drugs or for confirmed VF on first line ART [13]. Patients identified with VF on second line ART (>1000 copies/ml) after a minimum of six months of therapy, were referred for adherence counselling with a repeat VL measurement after three months.

### Inclusion and exclusion criteria

We identified HIV-infected adults over 18 years of age who initiated second line ART following VF on first line ART between August 2003 and June 2011. Patients who switched to second line ART for reasons other than VF on first line were excluded. In an exploratory analysis we discovered several patients with suppressed VL despite zero adherence, indicating that they were obtaining ART from another site. For the statistical analysis, we therefore excluded patients with suppressed VL following a four month period of null adherence.

Clinical and demographic data was extracted from the clinic’s electronic database. including age, sex, CD4 and VL responses, and duration on second line ART. The date of each pharmacy refill as well as the number of pills dispensed was also recorded. A 30 day supply of medication was routinely dispensed with pharmacy refills scheduled after 28 days. Stable patients with virologic suppression were occasionally given 60 or 90 days of medication with an appropriate follow up interval. Missed pharmacy refill dates resulted in a reminder telephone call from the clinic.

### Study design

We conducted a retrospective observational cohort study of patients who initiated second line ART after confirmed VF on first line. We analysed the relationship between short term adherence measured by pharmacy refill and virologic suppression. We identified factors associated with VF and evaluated three different short term measures of pharmacy refill adherence and compared them with long term adherence measured from second line ART initiation. In addition, we explored the optimum duration of short term refill which correlated best with a virologic response.

### Statistical analysis

We performed all statistical analyses in Stata version 13. The pharmacy refill period before each VL (after a minimum of 4 months on second line ART) was used to analyse the relationship between pharmacy refill adherence and virologic suppression. We ensured that our method accommodated patients who had collected more than 30 days of medication at a single visit within the period being assessed. The association between pharmacy refill adherence and virologic suppression was evaluated using Receiver Operator Characteristics (ROC). We compared several methods of calculating adherence from pharmacy refill data over differing periods. We assessed both short term (from 3 to 12 months) and long term pharmacy refill (from second line initiation). We truncated adherence over 100% for all methods.

Short term adherence was expressed as a percentage and calculated using three different methods, termed “interval gap”, “interval average” and “interval crude”. “Interval crude”, was calculated by dividing the number of pharmacy refills within the interval (numerator); by the number of months within the interval (denominator). “Interval average”, which is similar to a method published in a previous study on short term pharmacy refill adherence, [15] was calculated as follows: number of days of medication dispensed within the interval, plus the accrued days of medication from the last dispensing event prior to the interval, less the unused days of medication from the last dispensing event within the interval (numerator); divided by the number of days with the interval (denominator). “Interval gap” is a short term pharmacy refill adherence measure which accounts more accurately for gaps in medication days. The “interval average” method may over-estimate adherence, because patients receive 30 days’ supply at scheduled dispensing events every 28 days, therefore, if patients have a gap without medication but then attend subsequent scheduled dispensing events, the accrued two day supply for each dispensing event within the interval will be included in the numerator, reducing the days of medication missed. For each day within the interval, we determined whether the patients had medication according to the pharmacy refill data, allowing accrued tablets from the last dispensing event prior to the interval as with the “interval average” method. “Interval gap” was calculated as follows: the number of days within the period, less the number of days that the patient did not have medication (numerator); divided by the number of days within the period (denominator). Long term “overall” adherence was determined by dividing the total number of days of medication dispensed since second line initiation (numerator), by the total number of days since second line initiation prior to the VL of interest (denominator).

Associations between virologic suppression and the different adherence measures were determined using the area under the curve derived from receiver operating characteristic (ROC) analyses, assessed over differing interval durations prior to a VL of 3 to 12 months. The best performing short term adherence measure and interval duration prior to a VL was then selected for subsequent analyses. We determined the association between virologic suppression and the identified variables in a multivariate logistic regression model. The variables included age, sex, CD4 and log_10_ VL at second line initiation, duration on second line, and adherence measured by short term pharmacy refill prior to a VL. Missing CD4 and VL values at second line initiation were imputed. The square root of the CD4 was used to attenuate the effect of higher values.

### Ethics

This study was reviewed and approved by the University of Cape Town Human Research Ethics Committee and the McCord Research Ethics Committee.

## Results

Two hundred and ninety one patients met the inclusion criteria for the study, but there was missing data in 17 patients, who were excluded. The baseline characteristics of the 274 included patients are shown in Table 1. The proportions of patients with virologic suppression and VF over time on second line are shown in Table 2. For the subsequent analyses, we excluded 21 patients who had no VL data ≥4 months after starting second line and a further 10 patients who had suppressed VL following a 4 month period of zero adherence, leaving 243 patients.

**Table 1:** **Patient characteristics at initiation of second line ART (numbers in brackets = interquartile range)**

No of females	54.4% (149/125)
Median age in years	35 (32 – 42)
Median CD4 at baseline (n = 251)	174 (107 – 265)
Median log_10_ VL at baseline (n = 261)	4.1 (3.6 – 4.7)
Median no of months followed up	27 (15 – 47)

**Table 2:** **VL suppression over time**

	Months after starting second line							
	6	12	18	24	30	36	42	48
No of patients in care	252	228	180	146	112	87	72	54
No with VL results	223	197	160	124	97	78	69	45
No with VL <50	159 (71%)	155 (79%)	115 (72%)	97 (78%)	77 (79%)	58 (74%)	48 (70%)	33 (73%)
No with VL <400	195 (87%)	169 (86%)	132 (83%)	107 (86%)	88 (91%)	69 (88%)	60 (87%)	40 (89%)
No with VL ≥1000	22 (10%)	26 (13%)	26 (16%)	16 (13%)	7 (7%)	7 (9%)	2 (3%)	4 (8%)

Note that the 6 monthly VL data reflect a window (e.g. a VL between 9 and 15 months was categorised as a 12 month VL).

**Figure 1:**
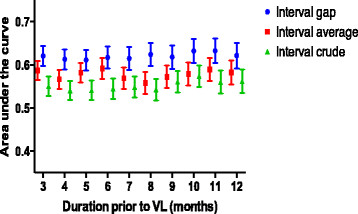
**Area under the receiver operator characteristics curve comparing “interval gap”, “interval average” and “interval crude” short term pharmacy refill methods over varying durations prior to the viral load of interest.** Error bars denote 95% confidence intervals.

**Figure 2:**
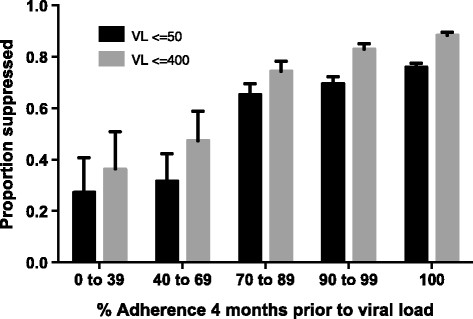
**Pharmacy refill adherence, measured by the “interval gap” method over 4 months, and virologic suppression.** Error bars denote upper 95% confidence intervals.

**Figure 3:**
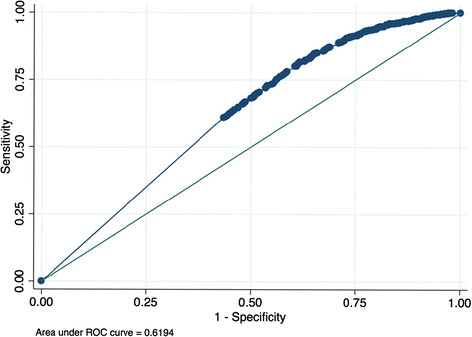
**Receiver operator characteristics curve illustrating the “interval gap” method over 4 months.**

Adherence measured by the “interval gap” method out-performed both the “interval average” and “interval crude” methods. (Figure 1) Adherence measured by the “interval gap” method performed similarly over all of the interval durations assessed, with overlapping 95% confidence intervals of the ROC area under the curve. We chose 4 months of adherence measured by the “interval gap” method for subsequent analyses as a pragmatic time period to implement in clinics, considering that achievement of virologic suppression on a new regimen is usually attained by 4 months. Short term pharmacy refill measured 4 months prior to a VL predicted virologic response, with higher rates of adherence achieving superior virologic suppression (Figure 2). The ROC curve for the “interval gap” method over 4 months duration is shown in Figure 3. Adherence measured by the “interval gap” method was equivalent to long term “overall” adherence at predicting virologic suppression (<400 copies/ml) – data not shown.

Significant risk factors for VF on multivariate analysis were poor adherence measured by 4 months of “interval gap” pharmacy refill proximal to a VL and a lower CD4 count at second line initiation (Table 3).

**Table 3:** **Factors associated with VF among patients on second line ART**

Variables		Univariate		Multivariate	
		Odds ratio	p-value	Adjusted odds ratio	p-value
		(95% CI)		(95% CI)	
**Adherence over 4 months (per 10% increase)**		−0.46 (−0.66 to −0.26)	<0.001	−0.73 (−1.34 to −0.12)	<0.001
**Time on second line ART**	**First year**	referent		referent	
	**After first year**	0.26 (−0.29 to 0.80)	0.358	1.05 (−0.12 to 2.23)	0.078
**Sex**	**Male**	0.72 (−0.21 to 1.66)	0.131	0.93 (−0.53 to 2.39)	0.214
	**Female**	referent		referent	
**Age (years)**	**<26**	1.7 (−2.16 to 5.57)	0.387	2.28 (−1.98 to 6.55)	0.294
	≥**26**	referent		referent	
**Log**_**10**_ **Viral load (copies/ml) at baseline**		0.31 (−0.26 to 0.87)	0.286	0.53 (−0.36 to 1.43)	0.244
**Square-root CD4 (cells/μL) at baseline**		−0.23 (−0.36 to −0.11)	<0.001	−0.22 (−0.35 to −0.09)	<0.001

## Discussion

We have demonstrated that short term adherence measured by pharmacy refill was the strongest predictor of VF on second line ART. Short term pharmacy refill adherence was also associated with virologic suppression with an “adherence dose response” relationship. Our study is one of few which have evaluated adherence on second line ART and is novel in that adherence was measured over the short term. We found that the “interval gap” method, which is a method not previously used for calculating adherence from ART pharmacy refills, outperformed the usual methods that average adherence over an interval. We observed a trend towards an association between longer duration of second line ART and risk for VF, but this was not statistically significant. We found an association between lower CD4 count at the time of starting second line ART and VF on second line ART, which adds support to data showing that second line outcomes are improved with early detection of failure of first line ART and prompt initiation of second line ART before immunological deterioration [18].

A previous report of an earlier cohort from the same clinic as the present study, [9] reported an association between virologic suppression on second line ART and adherence measured by pharmacy refill over the long term since second line ART initiation. Pharmacy refill as an adherence measure over shorter time periods is more pragmatic and implementable [19]. In our study short term “interval gap” refill performed similarly to long term “overall” refill on ROC analysis, and out-performed other methods of determining short term adherence using pharmacy refills. We explored the ability of differing durations of pharmacy refill from three to twelve months to predict VF: “interval crude” and “interval average” performed better with longer durations, but the best performing method, “interval gap”, performed similarly over all of the interval durations assessed. Grossberg et al. demonstrated that a 90 day period of pharmacy refill was associated with VL change, [19] but refill periods shorter than 60 days may overcall imperfect adherence leading to unnecessary clinical interventions [17]. We found that 80% adherence by pharmacy refill over 4 months appeared to be a threshold for predicting virologic suppression (Figure 2). However, there were small numbers of patients in the lower adherence strata, which limited our ability to determine a threshold. Others have reported an increased risk of VF with adherence <80% in observational studies of patients on boosted PI regimens [8],[20]. A threshold of 80% adherence measured by pharmacy refill in the previous 4 months could be used to identify patients needing enhanced adherence support and rationalise use of VL testing in resource-limited settings. Most patients on second line ART experiencing VF were able to achieve virologic suppression with intensified adherence support in a study at a clinic in South Africa [21]. VF on second line ART is likely a result of poor adherence rather than resistance as several studies have found a low proportion of major PI mutations in patients with VF on second line ART [10]-[12]. Unfortunately, as a result of high cost, the routine use of GART in patients with VF on second line will not be widely available in most low-middle income countries. Van Zyl et al. [12] suggested an algorithm to select patients in VF on second line for GART using lopinavir plasma and hair therapeutic drug monitoring. However, these pharmacokinetic measures are costly (although less costly than GART) and have extremely limited availability in resource-limited settings. By contrast, short term adherence measured by pharmacy refill can be easily implemented, especially in clinics with electronic dispensing, without incurring large additional costs.

Our study has several limitations. First, the rate of VF on second line in the McCord ART clinic was lower than reported in a recent systematic review of second line treatment outcomes in resource limited settings, possibly due to a high physician/nurse to patient ratio and reliable antiretroviral drug supply [2]. Therefore the findings may not be generalisable to public sector ART clinics in other settings. Second, pharmacy staff shortages in clinics with large patient numbers may be unable to apply the “interval gap” method to calculate adherence. However, the “interval crude” method, which is easy to calculate, could be instituted in pharmacies with staff shortages or those that only keep manual pharmacy refill records. Third, we excluded patients with zero adherence and suppressed VL on the grounds that they must have been collecting ART at another clinic. However, we had no way of determining this and it is possible that other patients may also have collected ART at other clinics, which would weaken associations between adherence and the virologic outcomes we assessed. Fourth, we lacked power for some of the associations we assessed, notably the duration of second line ART and risk for VF, and the determination of an adherence threshold for virologic suppression.

## Conclusion

In conclusion, short term pharmacy refill is an easily implementable adherence measure that can be used in ART clinics to identify patients at risk of VF on second line ART. Future studies need to be conducted in larger cohorts from clinics with a range of virologic outcomes in order to determine a threshold of adherence for predicting virologic response and to evaluate whether VL testing and GART could be limited to patients with better adherence.

### Consent

Written informed consent was obtained from each patient for the publication of this report and any accompanying images.

## Authors’ original submitted files for images

Below are the links to the authors’ original submitted files for images. Authors’ original file for figure 1 Authors’ original file for figure 2 Authors’ original file for figure 3 Authors’ original file for figure 5
