# Supplementary figures and images for: Short term adherence tool predicts failure on second line protease inhibitor-based antiretroviral therapy: an observational cohort study
Source: BMC Infect Dis. 2014 Dec 4;14:664. doi: 10.1186/s12879-014-0664-3 (PMC4266950; doi:10.1186/s12879-014-0664-3)

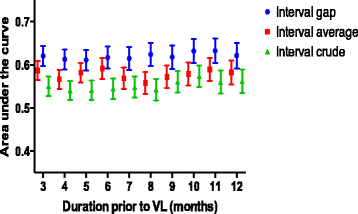

Supplement: Supplementary file 1 — Authors’ original file for figure 1 [file 12879_2014_664_MOESM1_ESM.gif]

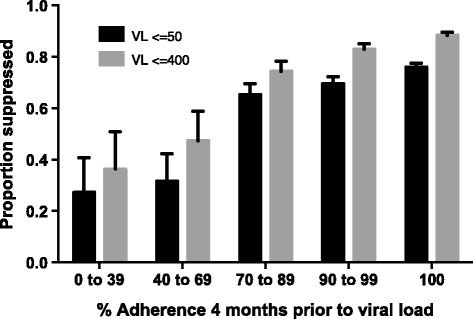

Supplement: Supplementary file 2 — Authors’ original file for figure 2 [file 12879_2014_664_MOESM2_ESM.gif]

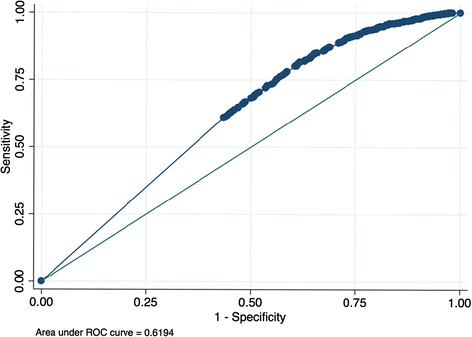

Supplement: Supplementary file 3 — Authors’ original file for figure 3 [file 12879_2014_664_MOESM3_ESM.gif]
