# Supplementary material for: Short term adherence tool predicts failure on second line protease inhibitor-based antiretroviral therapy: an observational cohort study
Source: BMC Infect Dis. 2014 Dec 4;14:664. doi: 10.1186/s12879-014-0664-3 (PMC4266950; doi:10.1186/s12879-014-0664-3)
Supplement: Supplementary file 4 — Authors’ original file for figure 5 [file 12879_2014_664_MOESM4_ESM.docx]

**TABLE 3: Factors associated with VF among patients on second line ART**

| **Variables** | | **Univariate** | | **Multivariate** | |
| --- | --- | --- | --- | --- | --- |
|  |  | **Odds ratio**  **(95% CI)** | **p-value** | **Adjusted odds ratio**  **(95% CI)** | **p-value** |
| **Adherence over 4 months**  **(per 10% increase)** | | -0.46 (-0.66 to -0.26) | <0.001 | -0.73 (-1.34 to -0.12) | <0.001 |
| **Time on second line ART** | **First year** | referent | | referent | |
|  | **After first year** | 0.26 (-0.29 to 0.80) | 0.358 | 1.05 (-0.12 to 2.23) | 0.078 |
| **Sex** | **Male** | 0.72 (-0.21 to 1.66) | 0.131 | 0.93 (-0.53 to 2.39) | 0.214 |
|  | **Female** | referent | | referent | |
| **Age (years)** | **<26** | 1.7 (-2.16 to 5.57) | 0.387 | 2.28 (-1.98 to 6.55) | 0.294 |
|  | **≥26** | referent | | referent | |
| **Log_10_ Viral load (copies/ml) at baseline** |  | 0.31 (-0.26 to 0.87) | 0.286 | 0.53 (-0.36 to 1.43) | 0.244 |
| **Square-root CD4 (cells/μL) at baseline** |  | -0.23 (-0.36 to -0.11) | <0.001 | -0.22 (-0.35 to -0.09) | 0.001 |
